# Supplementary material for: Cellular Stress Induces Nucleocytoplasmic Transport Deficits Independent of Stress Granules
Source: Biomedicines. 2022 May 3;10(5):1057. doi: 10.3390/biomedicines10051057 (PMC9138870; doi:10.3390/biomedicines10051057)
Supplement: Supplementary file 1 [file biomedicines-10-01057-s001.zip › biomedicines-1684023-supplementary.pdf]

Supplementary figures

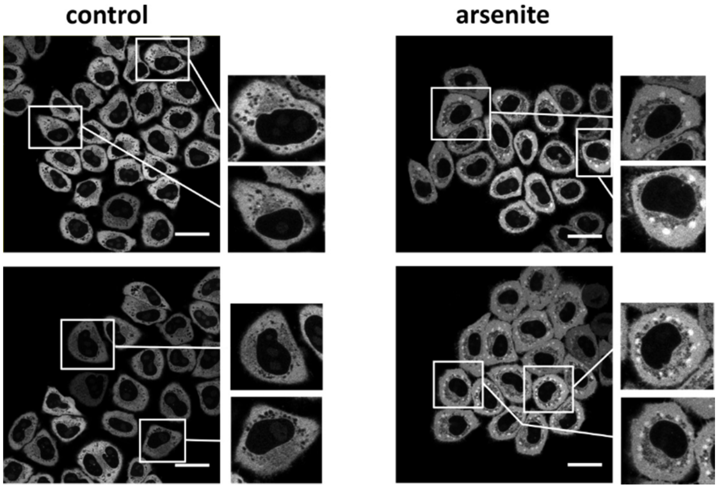

Supplementary Figure S1. Reporter localizes in stress granules. Representative images of NLS$_{SV40}$-mNeonGreen$_{2x}$-NES$_{pki}$ HeLa Kyoto cells. Cells were treated with arsenite for 1 h (H_2O as vehicle control) and fixed with 4 % PFA. No immunostaining was performed. The reporter localizes at cytoplasmic granules in the stress condition. Scale bar = 20 µm.

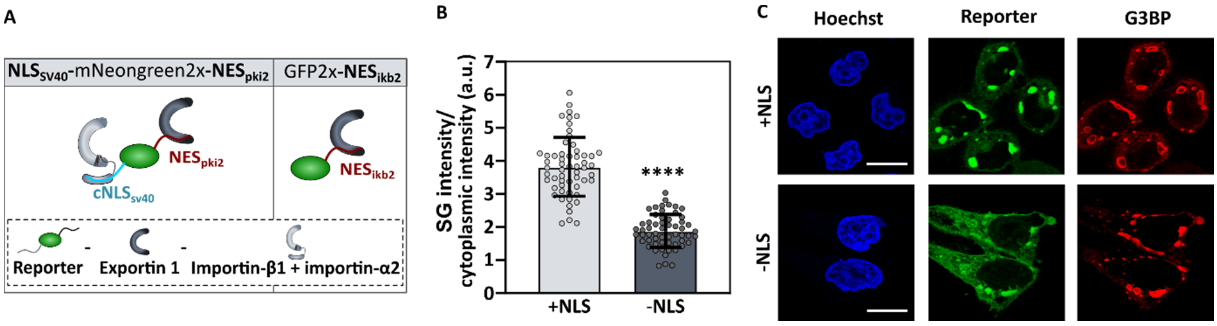

Supplementary Figure S2. **Importin-β1-mediated localization of the reporter at stress granules. (A)** HeLa Kyoto cells stably expressing NLS$_{SV40}$-mNeonGreen$_{2x}$-NES$_{pki}$ or GFP$_{2x}$-NES$_{ikb2}$. Only the former reporter binds to the importin-β1/α$_x$-complex, while both reporters bind to exportin 1. (**B)** The above cells were transfected with a G3BP1 plasmid and stained with an anti-G3BP antibody after fixation. Intensity of the reporter in stress granules compared to the cytoplasmic intensity was manually measured with the help of ImageJ. Each dot represents one cell, with n = 60 from three experiments. The GFP$_{2x}$-NES$_{ikb2}$ reporter was significantly less localized at stress granules. Unpaired t test. Means ± SD. **** indicates $p < 0.001$. **C.** Representative images are shown of NLS$_{SV40}$-mNeonGreen$_{2x}$-NES$_{pki}$ (+NLS) and GFP$_{2x}$-NES$_{ikb2}$ (-NLS) reporter cells overexpressing G3BP. Scale bar = 20 µm.

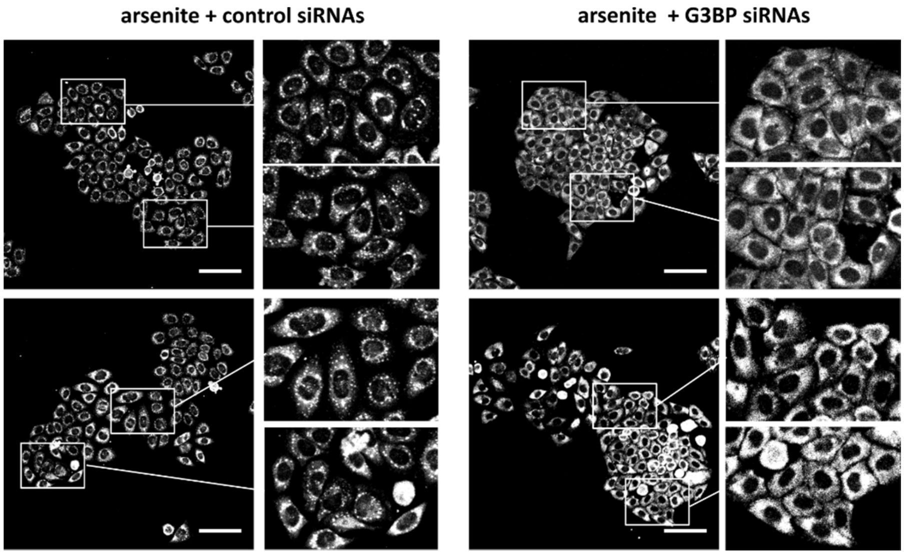

Supplementary Figure S3. Knock down of G3BP1 and G3BP2 using siRNAs inhibits the formation of stress granules after arsenite treatment. Representative images taken under the confocal microscope (96-well plate, 10 X, zoom 2X) of the same cells that were used to measure nucleocytoplasmic transport. Cells were stained with the stress granule marker yb1. Scale bar = 100 µm.

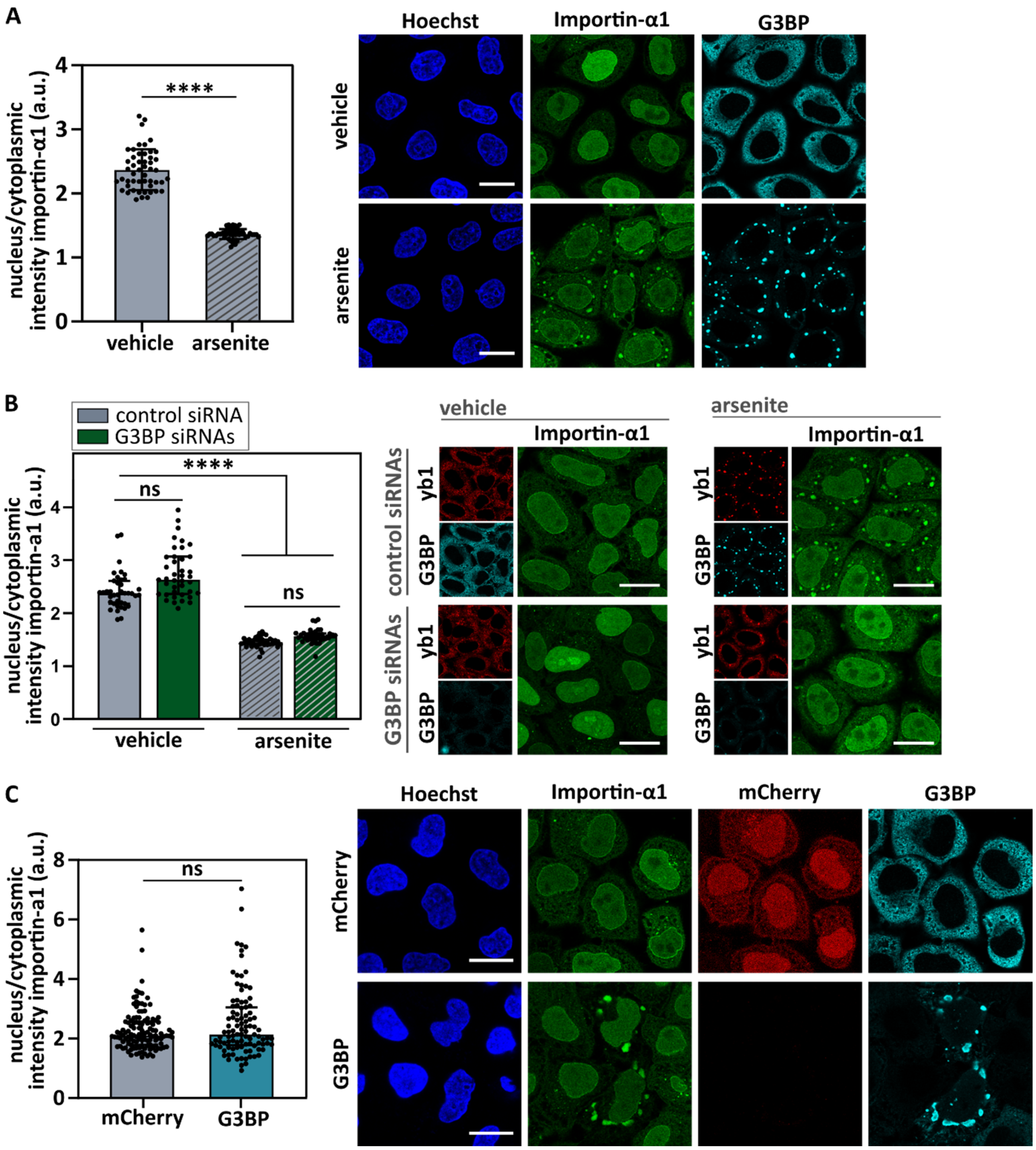

Supplementary Figure S4*.* **Stress granule-independent cytoplasmic localization of importin-α1. (A)** HeLa Kyoto cells endogenously expressing importin-α1-mNeonGreen were treated with arsenite, fixed and stained with an anti-G3BP antibody. A significantly increased cytoplasmic intensity was observed in arsenite-treated cells. Confocal images were automatically analyzed with CellProfiler. Dots represent means of one image with 10 - 50 cells per image; n = 40 from four experiments. Unpaired t test. Means ± SD. Scale bar = 20 µm. **** indicates $p < 0.001$. **(B)** HeLa Kyoto cells endogenously expressing importin-α1-mNeonGreen

were transfected with both G3BP1 and G3BP2-specific siRNA or control siRNA. After 48 h, cells were treated with arsenite, fixed and stained with an anti-G3BP and anti-yb1 antibody. Knock down of G3BP1 and G3BP2 did not rescue arsenite-induced increased cytoplasmic intensity of importin-α1. Confocal images were automatically analyzed with CellProfiler. Dots represent means of one image with 5 - 50 cells per image; n = 40 from four experiments. Kruskal-Wallis test followed by Dunn's multiple comparisons test. Median ± IQR. Scale bar = 20 µm. ns: no significant difference; **** indicates $p < 0.001$. (**C)** HeLa Kyoto cells endogenously expressing importin-α1-mNeonGreen were transfected with a plasmid expressing mCherry or G3BP1. After 48 h, cells were fixed and stained with an anti-G3BP antibody. No significant difference was observed in the nucleus/cytoplasmic ratio of importin-α1. Confocal images were manually analyzed with ImageJ. Each dot represents one cell with n = 100 (G3BP) or 120 (mCherry) from four experiments. Mann-Whitney U test. Median ± IQR. Scale bar = 20 µm. ns: no significant difference.

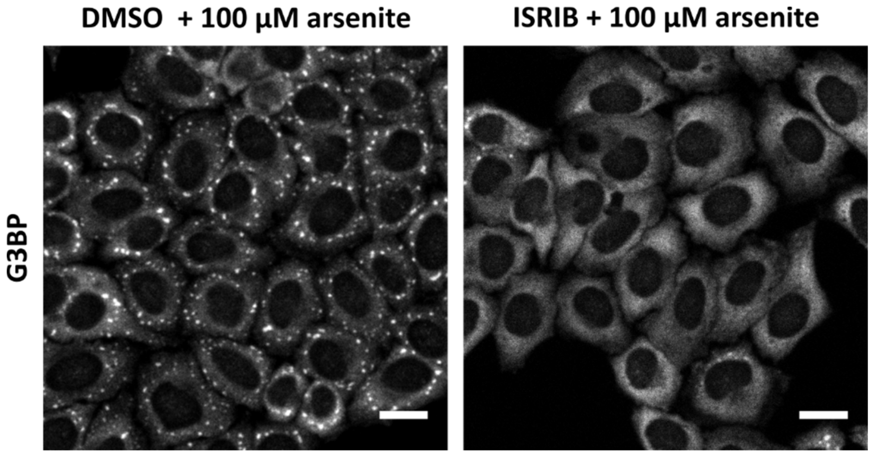

Supplementary Figure S5. Suppressing stress granule formation via inhibition of the integrated stress response Representative images taken under the confocal microscope (96-well plate, 10 X, zoom 7X) of the same cells that were used to measure nucleocytoplasmic transport. Cells were stained with the stress granule marker G3BP. Scale bar = 20 µm.

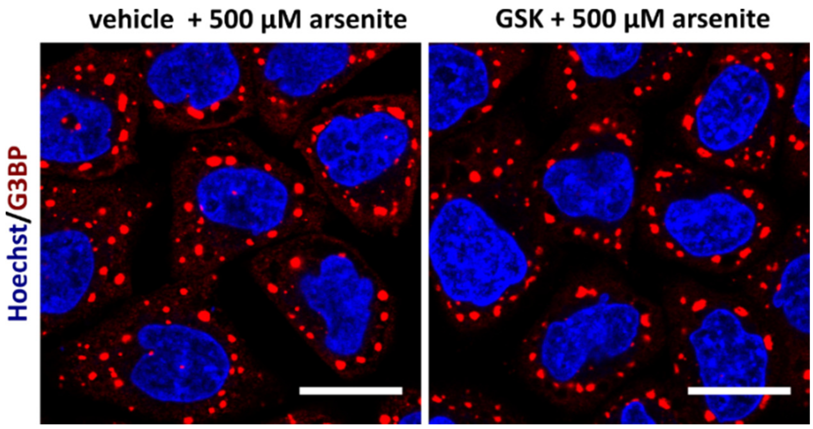

Supplementary Figure S6. The PERK inhibitor GSK2606414 does not inhibit arsenite induced stress granule formation. HeLa Kyoto cells were preincubated with 5 µM GSK2606414 (Sigma-Aldrich, 516535) for 4 h before treatment with 500 µM arsenite. After 1 h, cells were fixed with 4% PFA and stained with an anti-G3BP antibody. Scale bar = 20 µm.

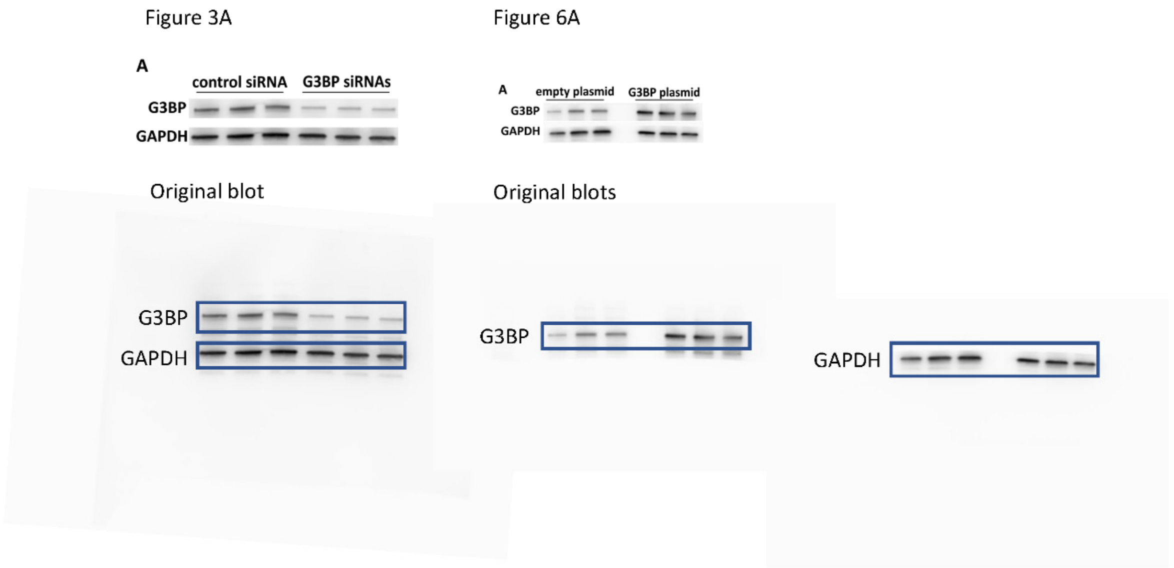

Supplementary Figure S7. Original blots. Uncropped versions of the blots used for the different Figures.
